# The Renin Angiotensin System: Insights into the Role of ACE2 in Glomerular Injury Including SARS-CoV-2 Infection

**DOI:** 10.3390/ijms27021033

**Published:** 2026-01-20

**Authors:** Everton Smith, James Scholey

**Affiliations:** 1 Department of Physiology, University of Toronto, Toronto, ON M5S 3K3, Canada; everton.smith@mail.utoronto.ca; 2 Department of Medicine, University of Toronto, Toronto, ON M5S 3H2, Canada

**Keywords:** angiotensin, angiotensin converting enzyme II, glomerulus, SARS-CoV-2

## Abstract

The circulating renin–angiotensin–aldosterone system (RAAS) plays a key role in regulating blood volume and electrolyte levels. While important for the maintenance of intravascular volume systemically, the local activation of tissue RAAS and the generation of angiotensin II contribute to inflammation and fibrosis. In the kidney, angiotensin II plays a key role in the development and progression of glomerular injury. Angiotensin-converting enzyme 2 (ACE2), an enzyme that degrades angiotensin II, is expressed in the glomerulus, focusing attention not only on the complexity of the RAAS but also identifying a potential new determinant of glomerular injury. Accordingly, we performed a narrative review using the search terms ACE2 and glomerulus in PubMed and Google Scholar to summarize the current understanding of the role of ACE2 in glomerular injury. We also discuss the role of ACE2 as a cellular receptor for SARS-CoV-2 and the potential impact of this function on glomerular injury in the setting of COVID-19.

## 1. Introduction

Our classical understanding of the RAAS is that it plays a dominant role in the regulation of blood volume, electrolyte balance, and the progression of CKD [1,2]. Renin, primarily in the afferent arteriole of the renal vasculature, is a proteolytic enzyme that plays a key role in activation of the RAAS. It is responsible for the conversion of angiotensinogen to angiotensin 1-10 (angiotensin I). Next, an enzyme called angiotensin-converting enzyme (ACE) cleaves two C-terminal amino acids from angiotensin 1-10 to produce angiotensin 1-8 (angiotensin II) [3]. Angiotensin II causes vasoconstriction of arterioles, aldosterone release from the zona glomerulosa of the adrenal cortex, and increased vasopressin release from the posterior pituitary gland. Aldosterone acts on the distal convoluted tubule of the nephron to increase Na^+^ reabsorption. Angiotensin II also directly increases Na^+^ reabsorption in the proximal tubule of the nephron. Vasopressin leads to the insertion of aquaporins onto the apical membrane of the collecting duct of the nephron. The resulting increase in collecting duct water permeability enhances water reabsorption down a concentration gradient. Taken together, vasoconstriction, in conjunction with aldosterone, direct tubular effects, and vasopressin, serves to maintain blood pressure, intravascular volume, and organ perfusion.

Activation of the intrarenal RAAS is one of the most common causes of CKD progression. This was demonstrated classically in studies of the remnant kidney model of experimental CKD in rats [4]. In this model, the development of glomerulosclerosis and proteinuria were attenuated by treatment with both ACE inhibition and angiotensin receptor blockade. These early studies focused attention on the intrarenal RAAS because measures of the circulating RAAS are suppressed. Subsequent studies showed that all of the components of the RAAS were expressed in the kidney including renin but also ACE, angiotensinogen, and the angiotensin II type 1 receptor (AT_1_R). Moreover, many studies have demonstrated that the effector of the RAAS, angiotensin II, stimulates the elaboration of extracellular matrix proteins that contributes to tissue fibrosis, as well as inflammation and the recruitment of monocytes and macrophages to the kidney [5].

Angiotensin II can impose these changes by binding two types of cell surface receptors: the angiotensin II type 1 receptor and angiotensin II type 2 receptor (AT_2_R). AT_1_R is a G protein-coupled receptor (GPCR), and its activation is closely linked to vasoconstriction as well as proinflammatory and profibrotic processes [6]. Following the activation of AT_1_R by angiotensin II, there are multiple downstream pathways that can be activated. Phospholipases A, C, and D are responsible for the production of arachidonic acid, phosphatidic acid, and inositol 1,4,5-triphosphate (IP3) and diacylglycerol (DAG), respectively. The production of IP3 and DAG can lead to Ca^2+^ and protein kinase C (PKC) activity, which triggers aldosterone release and vasoconstriction. Additionally, AT_1_R stimulation leads to an activation of the NADPH complex, which in turn produces reactive oxygen species, causing oxidative stress [7]. Recently, Xu et al. found that angiotensin II infusion in 6-week-old male C57BL/6 mice led to kidney injury characterized by an increase in serum creatinine and the albuminuria/creatinine ratio. This was accompanied by an increased expression of inflammatory cytokines like IL-1β, IL-6, and TNF-α in the kidney [8]. This experimental observation supports the hypothesis that activation of the RAAS contributes to the progression of CKD through AT_1_R-induced inflammation and fibrosis. Additionally, the vasoconstrictive effects of AT_1_R can elevate blood pressure, which can secondarily lead to progressive kidney injury.

Angiotensin II also binds and activates AT_2_R, which attenuates the intracellular effects of AT_1_R cell signaling, leading to vasodilation, reduced renin secretion, natriuresis, and lower blood pressure [9]. For many years, AT_2_R was felt to be the main way in which the tissue effects of angiotensin II and AT_1_R activation were counterbalanced. Similar to AT_1_R, AT_2_R is also a GPCR, and its activation is associated with the production of bradykinin, which elevates nitric oxide and cGMP levels [9]. Nitric oxide is a potent vasodilator. Additionally, AT_2_R activation was found to be associated with reduced renin synthesis and ultimately decreased angiotensin II production [10]. Finally, AT_2_R activation enhances natriuresis, which is the process of urinary Na^+^ excretion [11]. Due to the antihypertensive effects of AT_2_R, it has been identified as a potential reno-protective target. Supporting this, Kulkarni et al. found that in a high-sodium diet-fed rat model, the administration of an AT_2_R agonist prevented proteinuria [12].

Interestingly, the cellular effects downstream of the two receptor subtypes tend to counterbalance one another so that the overall effect of RAAS activation is dependent on relative expression levels of the receptor subtypes. It has been established that in the adult human kidney, only 5 to 10% of the angiotensin II receptors are AT_2_R, while the rest are AT_1_R [13]. This means that activation of the RAAS in the kidney is dominated by vasoconstriction, inflammation, and fibrosis, effects with important implications for kidney function and structure.

The discovery of another enzyme, angiotensin-converting enzyme 2 (ACE2), in the RAAS not only added an additional level of complexity to the RAAS, but also focused attention on the possible modulating role of the enzyme in kidney injury. Accordingly, the aims of this review are fourfold: (1) summarize the effect of ACE2 on the processing of angiotensin peptides; (2) given the importance of angiotensin II in glomerular injury, review the current understanding of the role of ACE2 in the development of glomerular injury, especially in the setting of diabetic glomerulopathy; (3) review the effect of angiotensin 1-7 and the activation of its cognate receptor, MasR, on glomerular injury; and finally (4) review the non-enzymatic role of ACE2 in glomerular injury secondary to SARS-CoV-2 infection.

## 2. ACE2 in the RAAS

ACE2 plays an important role in the production of effector peptides following RAAS activation. The gene for ACE2 is found on the short arm of the X chromosome, and it is composed of 18 exons [6]. It is expressed in many tissues, and it is highly expressed in the proximal tubular epithelial cells of the kidney [14]. It has also been shown that ACE2 is expressed differentially in males and females, with increased activity in females [15]. For example, studies from our laboratory group looked at ACE2 expression in the glomeruli of patients with CKD of varying etiology. In studies of healthy patients that were living kidney donors, ACE2 expression was greater in females than in males. ACE2 expression decreased in the tubulointerstitial compartment of the kidney with age but remained stable with age in the glomeruli [16]. We also observed that ACE2 expression was lower in the glomeruli of males with CKD than in females with CKD [17]. The mechanisms responsible for the impact of sex on ACE2 expression have not been fully elucidated, although some tissue studies suggest that there are correlations between the expression of ACE2 and estrogen receptor alpha. Other studies on the in vitro expression of ACE2 and cultured bronchial epithelial cells have shown that estrogen downregulates ACE2 expression. More work will be required to reconcile these in vivo and in vitro observations and to better understand the tissue-specific effect of sex hormones on ACE2 expression.

ACE2 is a membrane-bound carboxypeptidase with a metalloproteinase catalytic site at its extracellular N-terminal domain [18]. It contains a transmembrane domain and a cytoplasmic domain. ACE2 is capable of cleaving angiotensin 1-10 (angiotensin I) into angiotensin 1-9 and angiotensin 1-8 (angiotensin II) into angiotensin 1-7 (Ang-(1-7)). Thus, it plays a role in regulating tissue angiotensin II levels by degrading angiotensin II to Ang-(1-7).

**Figure 1:**
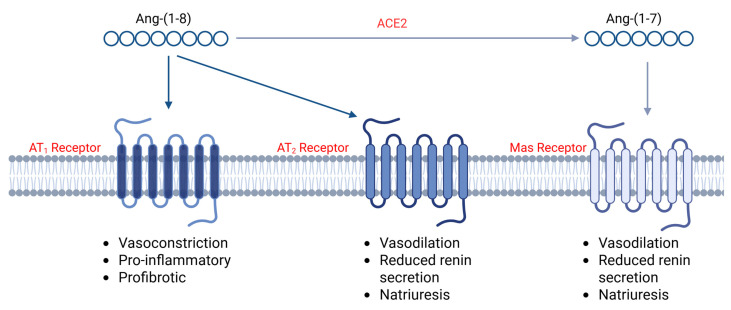
Diagram displaying the effects of AT_1_, AT_2_, and Mas cell surface receptor activation. Angiotensin 1-8 acts directly on both AT_1_ and AT_2_ receptors, while its metabolite, angiotensin 1-7 binds and activates Mas receptor. Created in BioRender. Smith, E. (2026) https://BioRender.com/s7n73g9 (accessed on 12 January 2026).

Ang-(1-7) is also an effector peptide of the RAAS, and studies show it has anti-inflammatory properties. A study by Zheng et al. showed that the administration of Ang-(1-7) in a high-fat diet (HFD)-induced renal injury model led to a significant improvement in inflammation, with decreases in the expression of TNF- α, IL-6, and MCP-1. This is due to Ang-(1-7) downregulating mRNA expression of low-density lipoprotein receptor (LDLr), sterol-regulatory element-binding protein-2 (SREBP2), and SREBP-cleavage activating protein (SCAP). Morphologically, an HFD led to a large glomerular size, tubular cell swelling, and brush border loss, all of which were prevented by Ang-(1-7) [19]. Ang-(1-7) engages the GPCR Mas, and the resulting transmembrane signaling also tends to counteract the effects of AT_1_R activation, as shown in Figure 1 [20]. The Mas receptor utilizes the RHO family of GTPases, ERK/MAPK, and PI3K/PRKD1/AKT/mTOR-mediated cell survival signaling pathways that contribute to normal cell survival and vasodilation [21].

ACE2 can be cleaved at the cell surface and released into the circulation or urine through a process called shedding. Although this form of soluble ACE2 is missing the transmembrane and cytoplasmic domain, it retains its enzymatic function [22]. A disintegrin and metallopeptidase domain 17 (ADAM17), also known as tumor necrosis factor α-converting enzyme (TACE), is responsible for cleaving the membrane-bound ACE2 [23]. PKC-δ is a critical upstream regulator of ACE2 shedding, and angiotensin II can also regulate ACE2 shedding by increasing TACE activity [24,25]. ACE2 shedding can be quantified by measuring ACE2 levels in the serum and urine, and early studies have focused on these measures in patients with diabetes. We reported that urinary ACE2 protein levels and activity were increased in the urine of subjects with uncomplicated type 1 diabetes mellitus during clamped euglycemia compared to healthy control subjects [26]. It is currently unclear whether the increased urinary ACE2 in subjects with diabetes reflects a deleterious effect of high glucose concentrations in the kidney, especially given there was no further increase in urinary ACE2 protein levels during clinical hyperglycemia [27]. It is tempting to speculate that the shedding of ACE2 by the renal tubular epithelium will be associated with a decrease in tissue ACE2 levels and therefore a decrease in the degradation of angiotensin II into smaller peptides like Ang-(1-7). At the present time, it is unclear if urinary ACE2 is a biomarker of RAAS activity, increased tissue angiotensin II levels in the kidney, or leads to higher rates of CKD progression.

## 3. ACE2 and Glomerular Injury

The discovery of ACE2 and its role in the metabolism of angiotensin peptides, specifically angiotensin II, led to the hypothesis that it could have protective effects against renal disease. Ye et al. set out to localize ACE2 expression in the renal tissue of control and diabetic mice with the use of immunofluorescence and confocal microscopy, as well as immunogold electron microscopy. Their studies revealed that ACE2 was expressed in the apical brush border of the proximal tubule and both visceral (podocytes) and parietal cells of the glomerulus. ACE2 was also found to be co-localized with proteins like nephrin, podocin, and synaptopodin, meaning it is present in the podocyte–slit diaphragm complex [28]. This is a dynamic cell junction connecting the foot processes of podocytes that plays an integral role in filtering large proteins [29]. In contrast, ACE was expressed in mesangial and endothelial cells but not within podocytes, suggesting that there was compartmentalized processing of angiotensin peptides in the glomerular capillary tuft [28].

Given the presence of ACE2 in glomerular podocytes, researchers have explored how the inhibition and deletion of ACE2 activity impacts glomerular structure and protein filtration. When Ye et al. inhibited ACE2 in a diabetic mouse model using MLN-4760, an ACE2 inhibitor, they saw a significant increase in albumin excretion in the mice with experimental diabetes compared to the vehicle group. They also saw an increase in fibronectin deposition in the glomerulus through immunohistochemistry of both diabetic and control model mice, a sign of glomerular injury [28]. This finding suggested that the loss of ACE2 activity accelerated diabetic kidney disease, at least in part due to increased kidney angiotensin II activity. In support of this hypothesis, the investigators showed that the increase in albumin excretion rate in the diabetic mice treated with the ACE2 inhibitor could be abrogated by concomitant treatment with an angiotensin receptor blocker. Taken together, these findings supported the notion that kidney angiotensin II levels in the murine diabetic kidney could be modulated by ACE2. In accordance with these experimental observations, subsequent studies by Reich et al. examined ACE and ACE2 mRNA levels in micro-dissected glomeruli and proximal tubules of patients with type 2 diabetes mellitus. They found that ACE2 mRNA levels were decreased in both compartments, while ACE RNA levels were increased, effects predicted to increase angiotensin II levels in the kidneys [30].

In the first studies of mice with a deletion of the gene for ACE2, there was a significant cardiomyopathy that developed in association with increased circulating angiotensin II levels as well as decreased circulating Ang-(1-7) levels. However, at three months of age, there was no evidence of kidney injury in the mice. The glomeruli were normal, with no evidence of mesangial matrix expansion or increased cellular proliferation. The tubular compartment and blood vessels within the kidneys did not exhibit any gross abnormalities. Male and female mice with a deletion in the gene for ACE2 were subsequently observed for up to 12 months. It was noted that there was an increase in the albumin excretion rate associated with glomerular injury developed in male mice. Light microscopic studies showed focal segmental glomerulosclerosis with evidence of hyalinosis and microaneurysm formation. Some mice exhibited diffuse marrow sclerosis, and electron micrographic studies showed the deposition of fibrillar proteins within the mesangial matrix. Immunohistochemical studies revealed increased type I and III collagen deposition in glomeruli, and the mesangial cells exhibited increased expression of alpha smooth muscle actin, suggesting a myofibroblastic phenotype. Additionally, there was evidence of increased oxidative stress likely downstream of angiotensin II. Glomerulosclerosis was attenuated by treatment with angiotensin receptor blockade. The emergence of late glomerulosclerosis in mice with a deletion of the ACE2 gene, with little large vessel or tubulointerstitial injury, indicated that the metabolism of angiotensin peptides by ACE2 within the microvascular glomerulus was important for the maintenance of normal structure and function [31].

Given the impact of pharmacologic blockade of ACE2 and deletion of the gene for ACE2 on glomerular injury, the next series of studies examined whether treatment with recombinant ACE2, or transgenic overexpression of ACE2 could influence the development of glomerular injury in murine models of CKD.

Oudit et al. were the first group to report that treatment with human recombinant ACE2 reduced the development of diabetic nephropathy in the Akita Mohs model of type 1 diabetes mellitus [32]. Recombinant ACE2 not only reduced blood pressure in this animal model of experimental diabetes, but also decreased the urinary albumin excretion rate. In addition, treatment with ACE2 attenuated mesangial matrix expansion in the glomerular capillary tuft and reduced the expression of type III collagen and alpha smooth muscle actin. These latter changes hearkened back to the observations made in studies of ACE2 gene deletion, which led to the development of a myofibroblastic-like phenotype in mesangial cells in association with the glomerular deposition of fibrillar collagens like type I collagen and type III collagen [31]. Interestingly, in vitro studies of mesangial cells showed that ACE2 reduced angiotensin II induced oxidative stress and the activation of any NADPH oxidase. These findings suggested that the cellular effects of angiotensin II could be aggregated by recombinant ACE2, likely due to degradation of the angiotensin II peptide limiting the activation of the AT_1_R on mesangial cells. The impact of recombinant ACE2 may also be due in part to an increase in Ang-(1-7) levels in the glomerulus. This is supported by the observation that Ang-(1-7) infusion decreased NADPH oxidase-induced oxidative stress, mesangial expansion, and the urinary albumin excretion rate in db/db mice, an experimental model of type 2 diabetes [32].

The studies of treatment with recombinant ACE2 have been complemented by studies with a genetic approach to increase ACE2 expression. The effects of ACE2 overexpression were first studied by Nadarajah et al. They utilized the nephrin promoter to generate a transgenic mouse that overexpressed ACE2 in glomerular podocytes [33]. Isolated glomeruli exhibited an increase in the mRNA and protein levels of ACE2. The investigators also measured ACE2 activity. Unlike previous studies utilizing a genetic mouse model with type 1 diabetes mellitus, this group administered streptozotocin to induce diabetes. They found an early increase in albuminuria in this mouse model, which was attenuated by overexpression of ACE2. This finding was analogous to the observations seen with recombinant ACE2 treatment, although the authors were careful to point out that the effect of port site overexpression of ACE2 on albuminuria was transient. Notwithstanding this observation, ACE2 overexpression reduced glomerular hypertrophy and mesangial matrix expansion and preserved the number of podocytes in diabetic mice. They also noted that there was a reduction in kidney transforming growth factor beta 1 (TGF-β1) expression, linking ACE2 overexpression to a reduction in the elaboration of extracellular matrix proteins driven in part by decreased TGF-β1. It is also possible that limiting angiotensin II’s activation of AT_1_R on podocytes limited the generation of reactive oxygen species and reduced glomerular apoptosis in the setting of diabetes, preserving the podocyte number in the glomerular capillary tuft and limiting changes in glomerular permselectivity.

Sanad et al. took a different approach to understanding the role of ACE2 in vascular disease. They overexpressed a human ACE2 construct driven by the muscle cell specific SM22α promoter in SD Hannover rats. This approach was associated with an increase in human ACE2 expression and activity in the kidney. SD Hannover rats develop spontaneous kidney damage at older ages. The damage is associated with the development of proteinuria and morphologic changes including mesangial matrix expansion and glomerulosclerosis. Urinary albumin and protein excretion rates were attenuated in the transgenic rats when they were assessed at 20–36 weeks of age. The reduction in proteinuria was associated with a reduction in protein casts in the renal tubules and decreased markers of tubular injury (neutrophil gelatinous-associated lipocalin (NGAL) and kidney injury molecular-1 (KIM-1)), and there was a decrease in glomerular injury. Rather unexpectedly, there were decreases in the mRNA levels for type I collagen, TGF-β1, and fibronectin [34]. These findings are important because they establish a role for ACE2 in the maintenance of normal glomerular structure and function with aging and support the earlier studies showing an increase in glomerular injury in older mice with a deletion in the gene for ACE2.

## 4. The Role of Ang-(1-7) in Glomerular Injury

The product of ACE2 enzymatic activity is Ang-(1-7). This peptide interacts with its cognate receptor, the Mas receptor (MasR), a GPCR. Silveira and coworkers gained some important insights into the effects of ACE2 by studying adriamycin-induced nephropathy targeting the podocyte [35]. The blockade of AT_1_R by losartan reduced kidney injury, but treatment with a MasR agonist also attenuated the histological changes downstream of adriamycin administration. These findings supported a role for Ang-(1-7) and MasR signaling in the protection of glomerular podocytes from injury. Interestingly, the adriamycin-induced injury was similar in wild-type mice and mice with a deletion of the MasR. The investigators concluded that the basal levels of Ang-(1-7) and MasR signaling were not protective at baseline. In separate studies of mice with a deletion in the gene for MasR, the deletion was associated with renal hyperfiltration and an increase in the urinary albumin excretion rate [36]. Extracellular matrix proteins like fibronectin and collagen were higher in the kidneys of mice with a deletion in the gene for MasR. This finding is not unlike the original observations in mice with a deletion of the gene for ACE2, suggesting that part of the impact of endogenous ACE2 on glomerular injury is due to an increase in Ang-(1-7) signaling via the MasR.

Recent studies suggest that Ang-(1-7) also signals through AT_2_R, complicating the interpretation of the previous studies. Studies performed in cultured human podocytes and in isolated glomeruli linked Ang-(1-7) to the generation of nitric oxide, with a subsequent influence on Ca^2+^ signaling [37]. In vitro studies of the interactions between angiotensin II and Ang-(1-7) in podocytes cultured in high glucose concentrations (30 mM) showed that glucose-induced decreases in nephrin, podocin, and WT1 expression, all markers of a differentiated podocyte, were attenuated by co-incubation with Ang-(1-7) [38]. The dedifferentiation of the cultured podocytes led to apoptosis, an effect also attenuated by Ang-(1-7) [38]. Second messengers downstream of the Ang-(1-7)/MasR include cAMP and phosphokinase A. The studies on vascular disease by Tetzner et al. suggest that there may be yet another receptor transducing the effect of Ang-(1-7) on cells called the MrgD receptor (Mas-related G protein-coupled receptor type D) [39]. The role of MrgD in kidney disease and renal homeostasis remains undefined, although a recent review suggests that the decarboxylation of the amino terminal aspartic acid of Ang-(1-7) generates a ligand for the MasR, and that receptor engagement opposes signal transduction activated by the AT_1_R [40].

Ang-(1-7) has a short half-life, so Cassis et al. studied the effect of administering a cyclic Ang-(1-7) with a longer half-life on kidney injury in Ob/Ob mice, an experimental model of type 2 diabetes mellitus [41]. This ligand attenuated albuminuria and reduced kidney inflammation even when added to treatments with an ACE inhibitor. This finding suggested that the cellular effects mediated by Ang-(1-7) were important in limiting kidney injury. This study confirmed earlier reports that Ang-(1-7) also limited kidney injury in rats with streptozotocin-induced diabetes mellitus, as well as in rats with experimental glomerulonephritis secondary to anti-Thy-1 antibody administration [42].

More recently, Klersy and coworkers reported that the AT_1_R-mediated attenuation of atherosclerosis in a Mohs model was dependent upon both ACE and Ang-(1-7) [43]. They showed that AT_1_R blockade was less effective in mice with a deletion of the gene for ACE and MasR. Taken together, the studies show that the blockade of the RAAS involves interacting peptides and their cognate receptors, and that both reduced angiotensin II and increased Ang-(1-7) at the cellular level play an important role in attenuating vascular injury, including injury in the glomerular capillary tuft [44].

Taken together, these above studies highlight that pharmacological inhibition, gene deletion, and gene overexpression support the hypothesis that ACE2 expression and activity play an important role in the maintenance of kidney health, likely on the basis of a reduction in local tissue angiotensin II levels. ACE2 not only protects against inflammation and histological changes to the glomerular mesangium, but it also maintains the integrity of podocytes and their functional role in glomerular permselectivity, in part due to a decrease in tissue angiotensin II levels and an increase in Ang-(1-7) levels.

Although the focus of these studies in the above sections is on the enzymatic activity of ACE2 and the resulting changes in tissue angiotensin peptide levels, it is now clear that ACE2 can function as more than an enzyme in the RAAS; it is also, rather unexpectedly, an important receptor.

## 5. ACE2 and SARS-CoV-2 Infection

**Figure 2:**
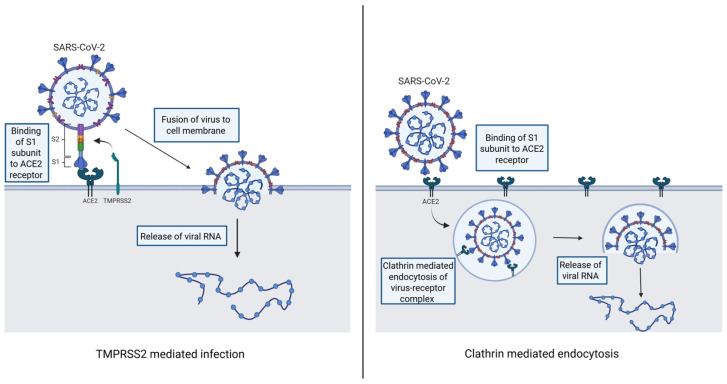
Diagram displaying the two pathways by which SARS-CoV-2 infects cells. Adapted from figure published by Jackson et al. [47]. Created in BioRender. Smith, E. (2026); https://BioRender.com/p96xb0f (accessed on 12 January 2026).

Almost 20 years ago, it was discovered that ACE2 was the cellular receptor for severe acute respiratory syndrome coronavirus 1 (SARS-CoV-1) and it is also the receptor for SARS-CoV-2, the virus responsible for the COVID-19 pandemic [45]. The SARS-CoV-2 virus contains a spike glycoprotein composed of homotrimers that transverse the virus membrane. The S1 subunit of the trimer has an N-terminal and receptor-binding domain that binds to ACE2 [46]. Binding of the virus to cell surface ACE2 is a critical first step to the internalization of the virus. Internalization proceeds through two pathways, as seen in Figure 2 [22,47]. In the first pathway, the S1 subunit spike protein binds the ACE2 receptor, which is then cleaved by transmembrane protease serine 2 (TMPRSS2). Following this cleavage, the virus fuses with the host cell membrane, and the viral mRNA is released into the cell. The second pathway is more common when there is limited expression of TMPRSS2 in the cell membrane. In this pathway, the S1 subunit of the virus binds ACE2, and the virus–ACE2 complex is internalized via clathrin-mediated endocytosis into endolysosomes. Following internalization, cathepsins cleave the S2′ site (internal component of S2 domain), and the endolysosome releases the viral mRNA [22,47].

Following internalization of the virus, ACE2 may undergo intracellular degradation in the lysosome, decreasing tissue ACE2, an effect that could promote inflammation and fibrosis [48,49]. A case–control study by Sultan et al. compared 50 healthy controls, 50 patients with moderate COVID-19 infection, and 50 patients with severe COVID-19 infection. An analysis of blood samples showed that patients with severe infection had elevated serum creatinine and urea levels, measures of acute kidney injury [50]. Similarly, the cohort analysis of patients with COVID-19 revealed decreased ACE2 expression in infected patients, along with increased urinary ACE2 levels when compared to non-COVID-19 controls, likely due to ACE2 shedding [51]. As previously described, ACE2 lowers angiotensin II levels and increases Ang-(1-7) levels, and by doing so protects the kidney from inflammation and glomerular damage. The loss of ACE2 due to SARS-CoV-2 infection could contribute to early renal inflammation and fibrosis following infection. Some studies suggest that severe COVID-19 infection is associated with acute kidney injury, and this and its relationship to CKD remain an area of active investigation [22].

Given that the entry of SARS-CoV-2 into the cell depends on both the spike protein and the membrane ACE2 enzyme, alterations to either of these factors can affect the ability of the virus to infect cells. The entry of SARS-CoV-2 into glomerular podocytes which express ACE2 and TMPRSS2 may have important implications for podocyte cell injury [16]. A study by Isnard et al. studied renal biopsies of patients with COVID-19. Of the 32 total biopsies, histological analysis revealed common patterns of COVID-19-associated kidney disease to be collapsing glomerulopathy (ten patients) as well as acute tubular injury (fourteen patients) and thrombotic microangiopathy (four patients) [52]. The relative prevalence of these histological findings were similar in other biopsy studies [53,54]. In collapsing glomerulopathy (CG), there is collapse of the glomerular tuft, along with hypertrophy and hyperplasia of podocytes [55]. It has been identified that, in the kidney, ACE2 and TMPRSS2 are highly expressed in tubule epithelial cells and podocytes [28]. Both entities are utilized in the viral entry of SARS-CoV-2, indicating the potential for the virus to directly induce CG. Clinically, this manifests in a nephrotic pattern of injury characterized by proteinuria. This phenomenon of SARS-CoV-2-induced CG is called COVID-19-associated nephropathy (COVAN).

A genetic predisposition to COVAN has been identified in patients with high-risk variants of the APOL1 gene (G1 and G2), common in those with African ancestry. The APOL1 gene is located on chromosome 22, and codes for a protein that protects against human African trypanosomiasis (sleeping sickness) caused by Trypanosoma brucei. The wild-type form is G0; however, the parasite adapted ways to evade trypanolysis, leading to positive selection of the G1 and G2 variants most commonly in those with Western African ancestry [56,57]. Molecularly, the G1 variant is a result of two amino acid substitutions near the C terminus, and G2 from a deletion of two amino acids [58]. Although beneficial for protection against parasitic infection, these alleles place individuals at risk of kidney disease, especially in the setting of a second hit such as COVID-19 infection. In a case series by Wu et al., six patients of African descent presenting with COVID-19 infection, proteinuria, and AKI had renal biopsies conducted. All these patients were identified to have collapsing glomerulopathy. APOL1 genotyping revealed that all patients had two high-risk alleles. An interesting finding was that SARS-CoV-2 RNA was not detected within the kidney during biopsy, leading to the proposal of a potential second hit hypothesis. The genetic predisposition paired with a viral infection causing cytokine storm might be responsible for this pattern of kidney injury [59].

The increased susceptibility of those with APOL1 variants prompted clinical trials to assess potential treatments. Patients with high-risk APOL1 genotypes and FSGS are typically treated with diuretics, RAAS inhibitors, and glucocorticoids, however this does not address the underlying genetic cause. Egbuna et al. are testing Inaxaplin to fill this gap for the treatment of proteinuric nephropathy in patients with high-risk APOL1 variants [60]. Inaxaplin has been shown in in vivo and in vitro studies to bind and inhibit APOL1 channel function, reducing proteinuria in a mouse model. In a phase IIa study assessing 13 patients with two APOL1 variants and either nephrotic or sub-nephrotic-range proteinuria and biopsy-proven FSGS, researchers found that those treated with Inaxaplin for 13 weeks had an average reduction in their urinary protein-to-creatinine ratio of 47.6%. This primary outcome represents a clinically significant decrease in proteinuria and highlights an area for future development for the medical management of proteinuric kidney disease in patients with APOL1 risk alleles.

It has been found that in the long term, previous SARS-CoV-2 infection can have negative impacts on kidney function. There is the potential for higher risks of AKI, eGFR decline, major adverse kidney events, and even end-stage kidney disease [61,62,63,64]. The prognosis of COVAN has not been studied in as much detail, highlighting a current gap in the literature. A retrospective case series looking at patients with COVAN found that 40 of 42 patients were diagnosed with CKD at follow-up (244 +/− 143 days) compared to 19 at baseline [65].

This pattern of kidney injury secondary to an infectious agent is reminiscent of HIV-associated nephropathy, termed HIVAN, which pathologically also caused collapsing glomerulopathy. HIVAN is caused by the viral infection of kidney cells by human immunodeficiency virus-1 (HIV). Similar to COVAN, the presence of APOL1 high-risk genotypes greatly increased the risk of HIVAN [66]. A cohort study followed 44 patients with confirmed HIVAN, comparing 28 who received treatment with fosinopril to 16 who refused this treatment and acted as controls. Wei et al. saw that all 16 who did not receive the ACE inhibitor progressed to end-stage renal disease (ESRD). This highlights the fact that if left untreated, HIVAN had the potential to progress to ESRD [67]. With increase in the use of highly active antiretroviral therapies, there has been a reduction in the progression to ESRD and need for dialysis in the HIVAN population [68].

As the pandemic progressed, variants of the SARS-CoV-2 virus emerged, such as delta and omicron. These variants are the result of genetic recombination, point mutations, deletions, and substitutions within genes for the virus that alter the coding regions for the spike protein. They can affect the receptor-binding domain of the virus and influence the affinity of the virus to the ACE2 receptor [69]. Another factor that influences the binding affinity of SARS-CoV-2 are the ACE2 receptors themselves. Researchers have investigated the potential genetic causes of increased susceptibility to SARS-CoV-2 infection based on single nucleotide polymorphisms (SNPs) for ACE and ACE2 genes. A common SNP in ACE2 is rs228566, which can have three possible genotypes: GG, GA, and AA. Early studies have shown that individuals with the GG genotype have a significantly increased risk of SARS-CoV-2 infection [70,71,72].

**Figure 3:**
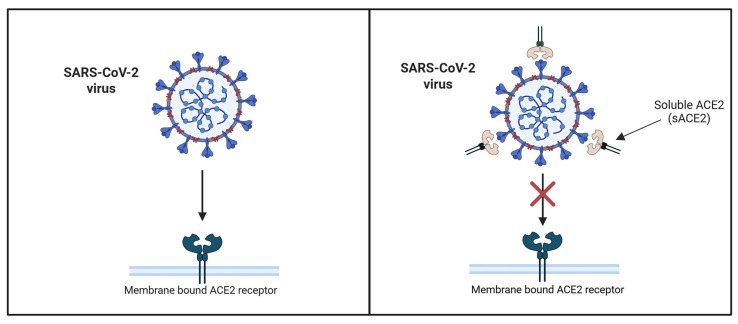
Soluble ACE2 acts as a decoy receptor to limit viral binding of SARS-CoV-2 to cellular ACE2. Image on the left depicting normal SARS-CoV-2 binding to cell surface ACE2. Image on the right depicts SARS-CoV-2 binding in the presence of soluble ACE2, preventing binding to cell surface ACE2.Adapted from figure published by Krishnamurthy et al. [80]. Created in BioRender. Smith, E. (2026); https://BioRender.com/fl8agss (accessed 12 January 2026).

Since the beginning of the COVID-19 pandemic, there has been a strong push to identify therapies to reduce susceptibility to the virus. After understanding the mechanism by which SARS-CoV-2 enters cells using ACE2, scientists have explored ways to prevent infection. One identified method is through the use of human recombinant human ACE2 (hrsACE2) [73]. Soluble ACE2 is not bound to the cell membrane and yet it retains the binding domain for SARS-CoV-2. The soluble ACE2 recombinant protein in theory could function as a decoy receptor limiting viral binding to the cellular ACE2, as seen in Figure 3. In studies of kidney organoids, Monteil et al. found that increasing concentrations of hrsACE2 decreased the level of SARS-CoV-2 infectivity [74]. A phase II double-blind, randomized, placebo-controlled trial assessed the safety of hrsACE2 in patients with COVID-19 and showed that the hrsACE2 treated group had a statistically significant decrease in viral load on day 3 and 5 of the 28 day trial [75]. Additionally, a case report on a 45-year-old woman showed that twice daily hrsACE2 infusion for 7 days led to a decrease in viral load, serum angiotensin II levels, and inflammatory cytokine levels [76]. These observations suggest that the recombinant protein served as a decoy receptor by binding the spike protein of SARS-CoV-2 and limiting interaction with endogenous membrane-bound ACE2. The maintenance of tissue ACE2 activity lowered angiotensin II and increased the anti-inflammatory Ang-(1-7) axis of the RAAS. A challenge with soluble forms of ACE2 is the relatively short half-life of about 10 h [77]. Studies on new approaches to the delivery of the recombinant ACE2 protein are currently underway. For example, vesicles have been explored as a method of delivery [73]. Recent research is also examining the use of antibodies and peptides that mimic the structure of soluble ACE2, retaining components like receptor-binding domains to act as decoy receptors for the SARS-CoV-2 virus [78,79].

## 6. Clinical Implications

There are several important clinical implications of the studies on ACE2 and Ang-(1-7) in the setting of glomerular injury. ACE2 activity leads to both a decrease in tissue angiotensin II levels and an increase in tissue Ang-(1-7) levels. The decrease in angiotensin II attenuates AT_1_R-mediated cellular effects on inflammation and fibrosis. Deletion of the ACE2 gene, abrogating the enzymatic activity, leads to an increase in age-related glomerulosclerosis in mice. Similarly, deletion of the MasR gene is also associated with age-related glomerular injury in mice, implicating a role for Ang-(1-7) in the maintenance of normal glomerular structure and function. Aging in humans is linked with the progressive development of glomerulosclerosis [81], and it is possible, but speculative, that variations in ACE2 expression and/or MasR expression in the glomerulus modulate age-related glomerulosclerosis in humans.

ACE2 and the MasR in the glomerulus have important clinical implications for diabetic nephropathy, a disease process that predominantly affects kidney glomeruli, as well as hypertensive glomerulopathy. Diabetes is associated with activation of the RAAS, and dual treatment approaches directed at blockade of the RAAS have beneficial effects on the outcomes of diabetic kidney injury [82]. The ongoing development of therapeutics related to the delivery of recombinant ACE2 proteins to the kidney and glomerulus may represent a future treatment approach to attenuating diabetic nephropathy and lowering blood pressure in humans. In addition to approaches to the delivery of ACE2 to the kidneys, the development of MasR peptide or non-peptide agonists may be another new treatment approach to diabetic nephropathy, akin to the approach taken with GLP1 receptor agonists [83].

Apart from clinical approaches based on enzymatic activity, the realization that ACE2 functions as the cell surface receptor for SARS-CoV-2, an essential first step towards internalization of the virus, has implications for treatment in humans even though COVAN is relatively uncommon. The development of ACE2-based peptides that serve as decoy receptors for viruses like SARS-CoV-2 may yield valuable treatment approaches to limiting tissue infection and injury by coronaviruses, reducing the overall severity of clinical infection. This may be particularly relevant for individuals with APOL1 high-risk alleles.

## 7. Conclusions

The regulation of tissue responses to RAAS activation is evolving as new components of the RAAS emerge. Studies show that both ACE2 activity and MasR activation maintain normal glomerular homeostasis and reduce glomerular injury, especially in the setting of experimental diabetic nephropathy, and these observations provide a rationale for new therapeutic approaches. Remarkably, the non-enzymatic function of ACE2 is also important because the protein is a SARS-CoV-2 receptor, supporting the development of novel therapeutics to limit this interaction. Taken together, ACE2 is a new therapeutic target that may play an important role in kidney disease ranging from the progression of CKD to viral infection.

## Data Availability

No new data were created or analyzed in this study. Data sharing is not applicable to this article.
